# A multicentric prospective observational study of diagnosis and prognosis features in ICU mesenteric ischemia: the DIAGOMI study

**DOI:** 10.1186/s13613-022-01092-8

**Published:** 2022-12-17

**Authors:** Simon Bourcier, Guillaume Ulmann, Matthieu Jamme, Guillaume Savary, Marine Paul, Sarah Benghanem, Jean-Rémi Lavillegrand, Matthieu Schmidt, Charles-Edouard Luyt, Eric Maury, Alain Combes, Frédéric Pène, Nathalie Neveux, Alain Cariou

**Affiliations:** 1 Medical Intensive Care Unit, AP-HP, Institut Cochin, Cochin Hospital, Centre & Université de Paris, INSERM U1016, CNRS UMR8104, 27 rue du Faubourg Saint-Jacques, 75014 Paris, France; 2 Assistance Publique-Hôpitaux de Paris, AP-HP, Médecine Intensive Réanimation, Pitié-Salpêtrière Hospital, Paris, France; 3 Clinical Chemistry Department, AP-HP Centre, Hôpital Cochin, Université de Paris, Paris, France; 4 EA 4466 PRETRAM, Faculty of Pharmacy, Université de Paris, Paris, France; 5 Réanimation Polyvalente, Hôpital Privé de l’Ouest Parisien, Ramsay Générale de Santé, Trappes, France; 6 INSERM U1018, Centre de Recherche en Epidémiologie et Santé des Populations, Team 5 (EpReC, Renal and Cardiovascular Epidemiology), Université Versailles Saint-Quentin, Villejuif, France; 7 AP-HP, Saint-Antoine Hospital, Assistance Publique-Hôpitaux de Paris, Médecine Intensive Réanimation, Paris, France; 8 INSERM UMRS_1166-iCAN, Institute of Cardiometabolism and Nutrition, Sorbonne Université, Paris, France

**Keywords:** Critically ill, Mesenteric ischemia, Observational, Plasma intestinal-fatty acid binding protein, Plasma citrulline

## Abstract

**Background:**

Non-occlusive mesenteric ischemia (NOMI) is a challenging diagnosis and is associated with extremely high mortality in critically ill patients, particularly due to delayed diagnosis and when complicated by intestinal necrosis. Plasma citrulline and intestinal-fatty acid binding protein (I-FABP) have been proposed as potential biomarkers, but have never been studied prospectively in this setting. We aimed to investigate diagnostic features, the accuracy of plasma citrulline and I-FABP to diagnose NOMI and intestinal necrosis as well as prognosis.

**Methods:**

We conducted a prospective observational study in 3 tertiary ICU centers in consecutive patients with NOMI suspicion defined by at least two inclusion criteria among: new-onset or worsening circulatory failure, gastrointestinal dysfunction, biological signs and CT-scan signs of mesenteric ischemia. Diagnosis features and outcomes were compared according to NOMI, intestinal necrosis or ruled out diagnosis using stringent classification criteria.

**Results:**

Diagnosis of NOMI was suspected in 61 patients and confirmed for 33 patients, with intestinal necrosis occurring in 27 patients. Clinical digestive signs, routine laboratory results and CT signs of mesenteric ischemia did not discriminate intestinal necrosis from ischemia without necrosis. Plasma I-FABP was significantly increased in presence of intestinal necrosis (AUC 0.83 [0.70–0.96]). A threshold of 3114 pg/mL showed a sensitivity of 70% [50–86], specificity of 85% [55–98], a negative predictive value of 58% [36–93] and a positive predictive value 90% [67–96] for intestinal necrosis diagnosis. When intestinal necrosis was present, surgical resection was significantly associated with ICU survival (38.5%), whereas no patient survived without necrosis resection (HR = 0.31 [0.12–0.75], *p* = 0.01).

**Conclusion:**

In critically ill patients with NOMI, intestinal necrosis was associated with extremely high mortality, and increased survival when necrosis resection was performed. Elevated plasma I-FABP was associated with the diagnosis of intestinal necrosis. Further studies are needed to investigate plasma I-FABP and citrulline performance in less severe forms of NOMI.

**Supplementary Information:**

The online version contains supplementary material available at 10.1186/s13613-022-01092-8.

## Introduction

Mesenteric ischemia represents one of the most life-threatening conditions affecting critically ill patients [1, 2]. Non-occlusive mesenteric ischemia (NOMI), defined by mesenteric ischemia without occlusion of major mesenteric arteries, is the main mechanism of mesenteric ischemia occurring in ICU setting [1, 3, 4]. In critically ill patients, NOMI is preceded by a state of acute circulatory failure and low mesenteric flow, resulting in digestive tract ischemic injuries, and may lead to diffuse transmural intestinal necrosis, associated with systemic inflammation, bacterial translocation, perforation and multiorgan failure. Thus, NOMI has been identified as a significant cause of death in ICU [4–10]. Despite intensive management including fluid resuscitation, organ supports, intravenous antibiotics and extensive intestinal resection of necrosed bowel segments, mortality remains very high, reaching 80% [1, 11, 12]. Furthermore, mesenteric ischemia related morbidity includes important functional , such as short bowel syndrome requiring total parenteral nutrition due to extensive intestinal resection [12, 13].

The lack of knowledge on NOMI physiopathology in critically ill patients together with a challenging and therefore often delayed diagnosis, are pointed out as the reasons for such a dismal prognosis [14, 15]. Clinical and biological anomalies lack sensitivity and specificity [16, 17]. Contrast-enhanced abdominal computed tomography (CT) scan, considered the cornerstone of mesenteric ischemia diagnosis [18], has a low negative predictive value and retrieved no sign of NOMI in one-quarter of patients with macroscopic NOMI diagnosis in a previously published cohort [4]. In this context, there is a need to have a better knowledge of time-course of intestinal failure and to identify an early, reliable and accurate diagnosis tool as could be a biological parameter. Specific bowel biomarkers such as plasma citrulline, an amino acid reflecting functional enterocyte mass, and intestinal-fatty acid binding protein (I-FABP), a cytosolic protein specific to small bowel released in case of ischemia, have been proposed but their performance remains to be evaluated especially in NOMI setting [19–21].

We aimed to prospectively investigate NOMI patients’ diagnostic features, the diagnostic accuracy of plasma citrulline and I-FABP in the absence and presence of intestinal necrosis as well as prognosis determinants.

## Study design and methods

### Study protocol

This prospective multicentric study was conducted between July 2016 and November 2018 in 3 medical-surgical ICUs located in tertiary university hospitals. Patients were enrolled in the study if NOMI was clinically suspected by physicians in charge in the presence of at least two inclusion criteria (Additional file 1) among:

- A new-onset or worsening circulatory failure,
- Digestive signs of gastrointestinal dysfunction [22],
- Biological signs evoking tissue ischemia,
- Contrast-enhanced abdominal CT-scan signs of mesenteric ischemia

Time of inclusion was defined by the starting of diagnosis process being the first confirmatory exam performed among CT, endoscopy or laparoscopy. Local investigators prospectively collected blood samples for biomarkers dosage at inclusion and collected in case report forms: demographics data, comorbidities, main diagnosis at ICU admission, organ supports at inclusion, worst values of last 24 h biological parameters and blood culture results, CT mesenteric ischemia signs, endoscopic and/or laparoscopic observations, definite NOMI or differential diagnosis retained after the diagnosis process, ICU survival and cause of death.

### Biomarkers measurements

Blood samples were collected and treated with centrifugation, aliquoted, and frozen at −80 °C at the study sites, then shipped to a central laboratory (Cochin Hospital) where the biomarkers assays were performed.

Plasma citrulline and arginine levels were measured by ion-exchange high-performance liquid chromatography (Aminotac, Jéol, Croissy-sur-Seine). Plasma I-FABP concentrations were assessed by ELISA according to the manufacturer's specifications (R&D Biotech, Minneapolis, MN). The lower threshold for I-FABP quantification was 15.6 pg/mL^−1^. Clinicians were blinded for biomarker levels results.

### Definitions of gastrointestinal failure, NOMI and intestinal necrosis

Gastrointestinal failure was defined by the presence of clinical and/or CT-scan signs of gastrointestinal dysfunction associated with circulatory failure [14]. Patients were classified as having confirmed or ruled out NOMI according to CT, endoscopy, and/or surgery findings. CT patterns of NOMI included bowel wall thickening or thinning with distension, lack of parietal enhancement, parietal pneumatosis and portal gas. Gastrointestinal endoscopy included gastroscopy, rectosigmoidosopy and colonoscopy, for which three grades were distinguished: grade 1, mucosal edema and erythema; grade 2, non-necrotic ulcerations on an oedematous mucosa; and grade 3, necrosis with grey–black mucosal discoloration. Necrosis was defined by endoscopic (grade 3) or surgical evidence of gastrointestinal necrosis. NOMI at the stage of ischemia was defined by CT, endoscopic findings grade 1 to 3 or surgical findings.

Our methodology for NOMI diagnosis exclusion was previously described [10] and is based on the following considerations. First, CT lacks sensitivity [4], and secondly, some distal lesions may be inaccessible by endoscopy. Therefore, NOMI at the stage of ischemia or necrosis was considered excluded in patients with two negative confirmatory investigations **(**Additional file 1**)**.

### Statistical analyses

Continuous variables (expressed as median [interquartile range, IQR]) were compared with Student’s *t*-test or the Wilcoxon test, as appropriate. Categorical variables (expressed as number (%)) were compared with χ^2^ or Fisher’s exact tests. Patients’ demographic, clinical features, laboratory results, CT findings and management characteristics, were tested in bivariate analyses for association with digestive ischemia or necrosis diagnosis. The ability of biomarkers to discriminate between definite and ruled out intestinal necrosis was investigated using the area under the receiver operating characteristic curve (AUROC) analysis. Cut-off values were set maximizing the Youden’s index.

Lastly, a univariable and multivariable Cox cause-specific proportional hazards model was used to investigate the association of clinical, biological and CT features with ICU mortality. Variables associated with ICU mortality (*P* < 0.05) were then included in the multivariable model and selected in the final model using a backward-stepwise variable elimination process. Kaplan–Meier survival curves were plotted for subgroup of patients with ruled out and definite digestive necrosis according to treatment by necrotic bowel segments resection.

*P* < 0.05 defined significance and no missing data imputation was made. Analyses were computed with R software, version 3.5.2 (https://www.r-project.org).

## Results

### Study population

**Fig. 1:**
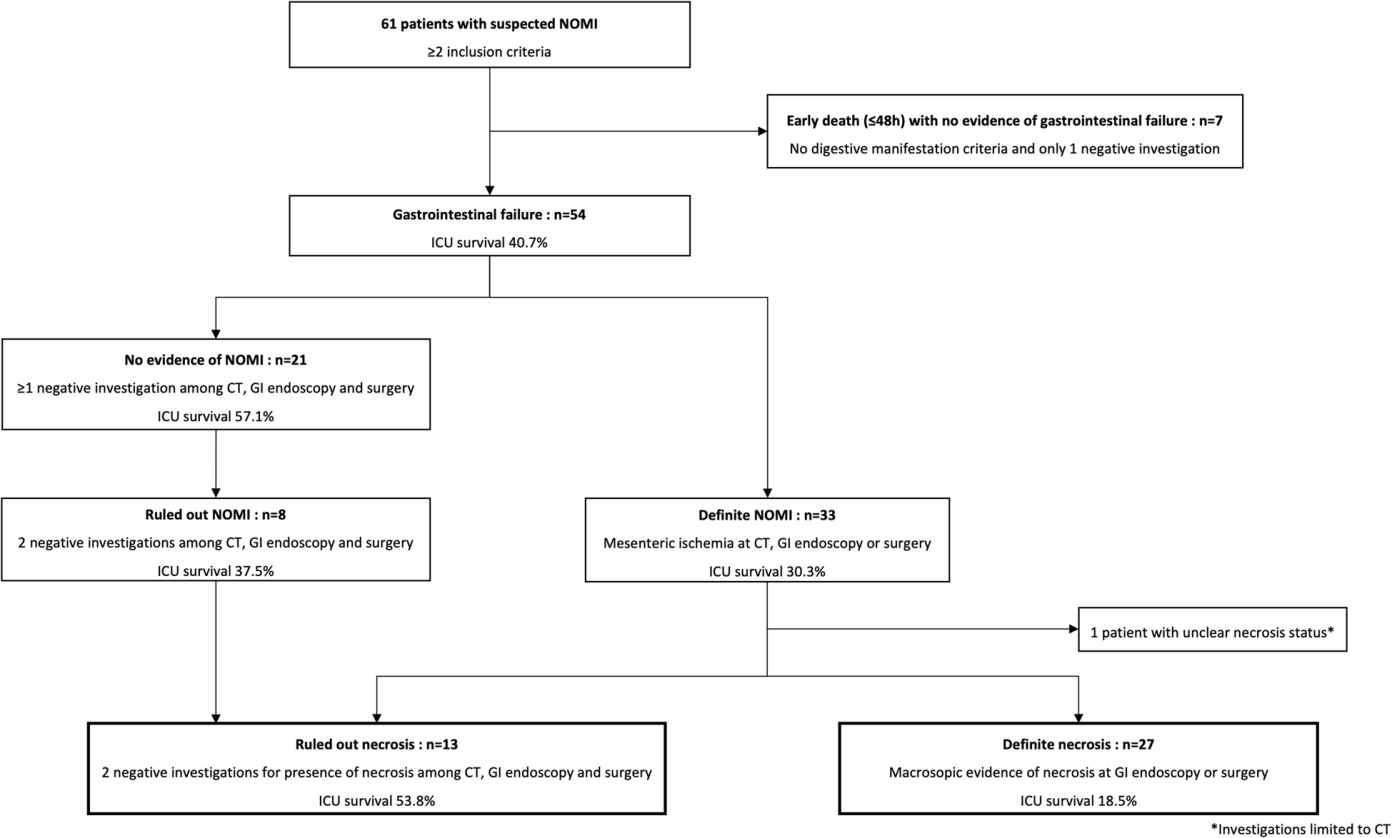
Flowchart of patients’ classification according to the presence or absence of NOMI and necrosis

A total of 61 patients with NOMI suspicion were included over the 2-year study period (Fig. 1, Additional file 1: Table S1). Patients were predominantly men (62%), aged in median [IQR] 64 years [57–69], with severe condition at admission as reflected by Simplified Acute Physiology Score (SAPS) II (71 [54–77]) and SOFA score (12 [9–16]). The most frequent diagnosis at admission were cardiogenic shock (33%), followed by septic shock (25%).

At inclusion, median time from ICU admission was 5.7 days [1.6–10.6]. Overall, patients suffered multiorgan failures as assessed by high SOFA score values (16 [11–19]), and required multiple organ supports: mechanical ventilation (84%), renal replacement therapy (71%), norepinephrine (median dose 0.72 µg/kg/mn [0.20–1.93]) and venous arterial ECMO (34%).

Using definition criteria for NOMI and intestinal necrosis, 54/61 patients were classified having gastrointestinal failure, NOMI was definite for 33 patients and ruled out for 8 patients, and intestinal necrosis was diagnosed for 27 patients and ruled out for 13 patients (Fig. 1). Among 27 patients with definite necrosis, necrosis was not clearly confirmed by investigations for 3 patients but was considered highly probable due to early death without differential diagnosis and observed gastrointestinal ischemia at CT or endoscopy.

Necrosis could reach all gastrointestinal tract and was extensive (≥ 2 locations) in 71% (Additional file 1: Table S7).

### Clinical, routine biological and CT parameters associated with NOMI and intestinal necrosis

Digestive signs presented poor sensitivity and specificity for diagnosis of NOMI and intestinal necrosis (Table 1, Additional file 1: Table S3). Arterial lactate, aspartate transaminase, alanine transaminase, LDH and CPK were significantly increased in patients with definite NOMI and intestinal necrosis compared with patients having further ruled out diagnosis, whereas procalcitonin was solely significantly increased in patients with intestinal necrosis.

**Table 1:** Digestive, biological parameters and CT results among patients with defined and ruled out intestinal necrosis

Characteristics	Gastrointestinal failure (*n* = 54)	Ruled out necrosis (*n* = 13)	Definite necrosis (*n* = 27)	*P *value*
Digestive manifestation criteria	47 (87.0)	12 (92.3)	23 (85.2)	1
Upper digestive hemorrhage	4 (7.4)	1 (7.7)	2 (7.4)	1
Vomiting or gastric residual**	23 (42.6)	6 (46.2)	12 (44.4)	1
Lower digestive hemorrhage	15 (27.8)	7 (53.8)	8 (29.6)	0.17
Diarrhea	31 (55.6)	8 (61.5)	13 (48.1)	0.51
Abdominal pain	22 (40.7)	3 (23.1)	10 (37.0)	0.48
Abdominal distension	17 (31.5)	2 (15.4)	13 (48.1)	0.08
Worst 24 h prior inclusion biological findings				
Leukocyte count (G/L)	16.6 [9.8–24.9]	10.3 [8.3–19.2]	20.1 [10.0–26.4]	0.16
Platelets (G/L)	107 [56–228]	124 [73–214]	78 [44–107]	0.09
Arterial lactate, mmol/L	5.2 [3.0–8.3]	4.0 [2.8–7.9]	6.6 [3.9–12.9]	0.05
Arterial pH	7.32 [7.24–7.43]	7.40 [7.29–7.47]	7.31 [7.13–7.38]	0.02
Bicarbonates, mmol/L	18.9 [13.6–21.5]	20.5 [19.1–23.2]	14.5 [12.2–19.4]	0.01
Potassium, mmol/L	4.6 [3.9–5.3]	4.3 [3.6–5.0]	5.0 [4.5–5.4]	0.09
Aspartate transaminase (IU/L)	175 [65–819]	66 [43–196]	358 [105–1731]	0.02
Alanine transaminase (IU/L)	123 [50–549]	55 [37–115]	346 [109–778]	0.01
Bilirubin (µmol/L)	34 [13–123]	21 [16–61]	65 [22–162]	0.14
LDH (IU/L)	757 [436–2366]	510 [367–784]	1822 [779–4150]	0.003
CPK (IU/L)	770 [149–2543]	359 [105–974]	1794 [864–8683]	0.01
Procalcitonin (µg/L)	6.4 [2.0–15.5]	0.7 [0.4–4.0]	11.9 [5.7–24.0]	0.005
Positive blood culture	14 (25.9)	2 (15.4)	8 (29.6)	0.45
Candidemia	4 (7.4)	0 (0.0)	3 (11.1)	0.54
Specific biomarkers				
Plasma I-FABP (pg/mL)	2976 [1143–9493]	1137 [639–2130]	6925 [2100–29686]	0.001
Plasma citrulline (µmol/mL)	20 [13–29]	28 [15–42]	19 [16–29]	0.61
Plasma arginine (µmol/mL)	43 [29–63]	53 [39–64]	43 [29–73]	0.38
Plasma citrulline/arginine ratio	0.46 [0.27–0.68]	0.44 [0.25–0.69]	0.47 [0.36–0.63]	0.46
CT conclusion				
Not done	9 (16.7)	0 (0.0)	7 (25.9)	0.08
No sign of mesenteric ischemia	29 (53.7)	11 (84.6)	6 (22.2)	< 0.001
Mesenteric ischemia	16 (29.6)	2 (15.4)	14 (51.9)	< 0.001
CT findings				
Abnormal wall enhancement	14 (31.1)	2 (15.4)	11 (55.0)	0.03
Pneumatosis intestinalis	4 (8.9)	0 (0.0)	4 (20.0)	0.14
Bowel dilation	16 (35.6)	3 (23.1)	11 (55.0)	0.09
Portal venous gas	2 (4.4)	0 (0.0)	2 (10.0)	0.51
Atherosclerosis of mesenteric arteries	13 (29.5)	4 (30.8)	6 (30.0)	1

*CT* computed tomography

Results are expressed as median [interquartile range] or *n* (%). *P-value is presented for statistical comparison of ruled out and definite necrosis. **Residual gastric volume was considered if ≥ 300 mL.

Regarding CT performance for diagnosis of intestinal necrosis, sensitivity for abnormal wall enhancement and bowel dilation observation were 55.0%, 19.0% for pneumatosis intestinalis and 10.0% for portal venous gas (Table 1). Interestingly, CT scan found none of these signs in 30% of patients with intestinal necrosis.

### Accuracy of biomarkers for intestinal necrosis diagnosis

**Fig. 2:**
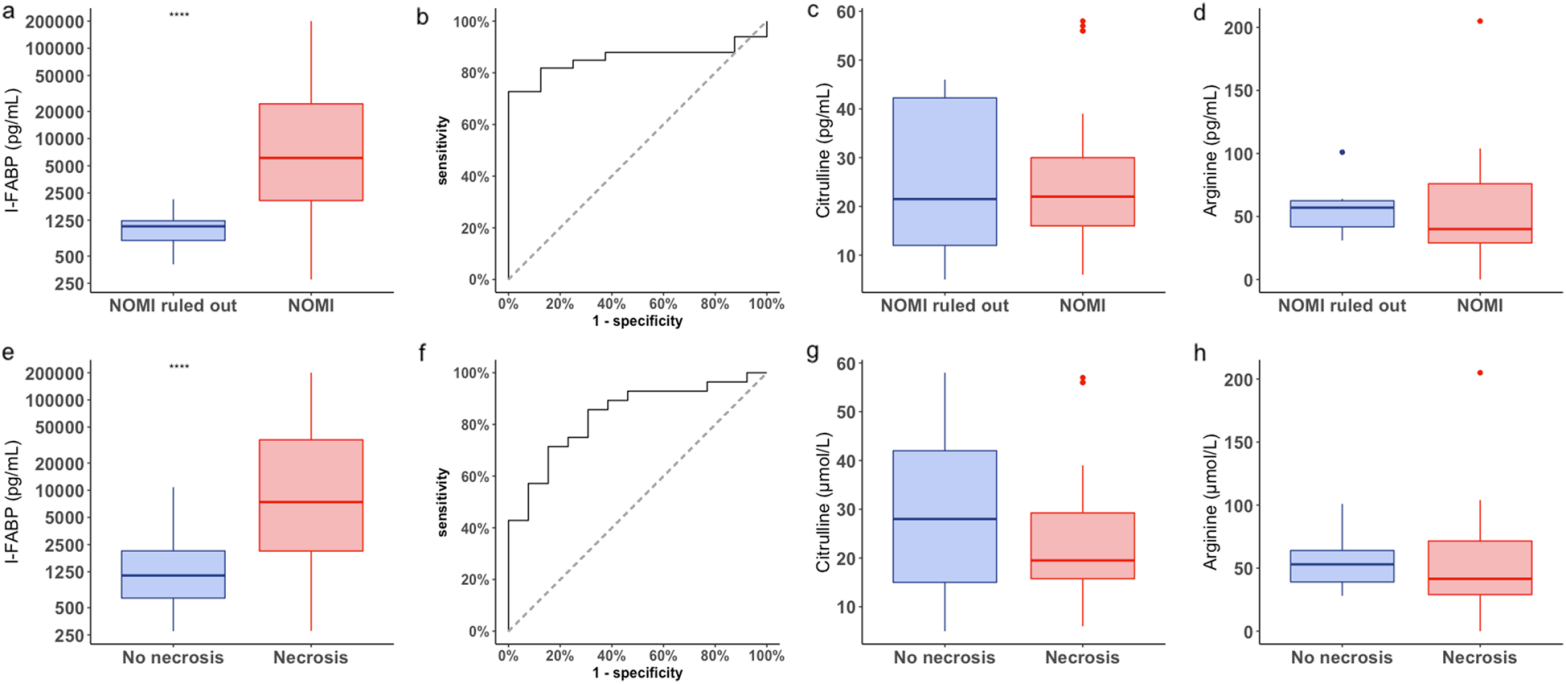
Boxplot (**a**) and ROC curve (**b**) of plasma I-FABP, boxplot of plasma citrulline (**c**), plasma arginine (**d**) for intestinal necrosis diagnosis. Box plot (**e**) and ROC curve (**f**) of plasma I-FABP, box plot of plasma citrulline (**g**) and plasma arginine (**h**) for NOMI diagnosis

Median plasma I-FABP concentration was significantly higher in patients with definite intestinal necrosis compared to patients with ruled out diagnosis: 6925 pg/mL [2100–29686] versus 1137 pg/mL [639–2130] (*p* = 0.001) (Fig. 2, Table 1). No relationship was noticed between plasma citrulline or arginine concentrations and presence of intestinal necrosis.

AUC of plasma I-FABP concentration for intestinal necrosis diagnosis was 0.83 [0.70–0.96]. Plasma I-FABP at the threshold of 3114 pg/mL presented a sensitivity of 70% [50–86], specificity 85% [55–98], negative predictive value 58% [36–93], positive predictive value 90% [67–96], positive likelihood ratio 4.57 [1.25–16.75] and negative likelihood ratio 0.35 [0.19–0.65] (Fig. 2).

### Outcomes associated with definite NOMI and intestinal necrosis

Patients with intestinal necrosis presented positive bacterial blood cultures in 29.6%, candidemia in 11.1% and peritonitis in 30% (Table 1, Additional file 1: Table S7).

ICU survival was 30.3% in patients with NOMI and 18.5% in patients with intestinal necrosis. Age was significantly associated with ICU mortality and ICU survivors required significantly lower dose of norepinephrine at time of diagnosis of NOMI and necrosis compared to non-survivors (Table 2, Additional file 1: Tables S4, S5 and S6).

**Table 2:** Cox proportional hazards model univariable and multivariable analysis of ICU mortality-associated factors in patients with definite intestinal necrosis

Characteristics of necrosis population	Univariate analysis			Multivariate analysis		
	CSH	95% CI	*P value*	CSH	95% CI	*P value*
Age (+ 10 years)	1.32	0.92–1.90	0.13	1.67	1.01–2.80	0.04
Males	1.44	0.61–3.39	0.41	–	–	–
Body mass index (kg/m^2^)	0.99	0.92–1.06	0.74	–	–	–
Diabetes	0.86	0.20–3.71	0.85	–	–	–
Hypertension	0.55	0.23–1.31	0.18	–	–	–
Smoking	1.19	0.51–2.77	0.68	–	–	–
Coronary disease	0.69	0.27–1.79	0.45	–	–	–
Peripheral arterial disease	0.66	0.15–2.83	0.57	–	–	–
End stage renal disease	2.06	0.27–16.2	0.49	–	–	–
Atrial fibrillation	0.77	0.26–2.28	0.63	–	–	–
Abdominal distension	2.47	1.05–5.86	0.04	4.99	1.62–15.3	0.005
SOFA at day of suspicion	0.99	0.87–1.13	0.93	–	–	–
Biological parameters						
Lactate (mmol/L)	1.10	1.02–1.19	0.03	1.17	1.05–1.30	0.005
pH (+ 0.1 unit)	0.69	0.50–0.94	0.02	–	–	–
Bicarbonates	0.97	0.89–1.06	0.52	–	–	–
LDH (+ 300 units)	1.02	0.99–1.06	0.21	–	–	–
CPK (+ 1.000 units)	1.001	0.98–1.02	1	–	–	–
Aspartate transaminase (+ 100 units)	1.001	0.98–1.02	0.94	–	–	–
Procalcitonin (µg/L)	0.97	0.89–1.06	0.52	–	–	–
Positive blood cultures	0.93	0.36–2.37	0.87	–	–	–
Candidemia	1.50	0.43–5.22	0.52	–	–	–
Specific biomarkers						
Plasma I-FABP (+ 1.000 units)	1.02	1.01–1.03	0.02	1.00	0.99–1.02	0.67
Citrulline (µmol/mL)	1.02	0.98–1.05	0.32	–	–	–
Arginine (µmol/mL)	1.01	0.99–1.02	0.09	–	–	–
Plasma citrulline/arginine ratio	1.06	0.54–2.08	0.87	–	–	–
CT findings						
Abnormal wall enhancement	0.93	0.34–2.48	0.87	–	–	–
Pneumatosis intestinalis	1.54	0.43–5.48	0.50	–	–	–
Bowel dilation	0.76	0.28–2.05	0.59	–	–	–
Portal venous gas	0.30	0.04–2.32	0.25	–	–	–
Atherosclerosis of mesenteric arteries	0.89	0.29–2.79	0.85	–	–	–
Organ supports at inclusion						
Norepinephrine (μg/kg/mn)	1.20	1.03–1.41	0.01	–	–	–
Mechanical ventilation	3.07	0.41–23.0	0.27	–	–	–
Renal replacement therapy	1.10	0.32–3.74	0.88	–	–	–
Surgical treatment						
Resection	0.31	0.12–0.75	0.01	0.49	0.18–1.33	0.16

*CSH* cause-specific hazard ratio, *SOFA* Sequential Organ-Failure Assessment

**Fig. 3:**
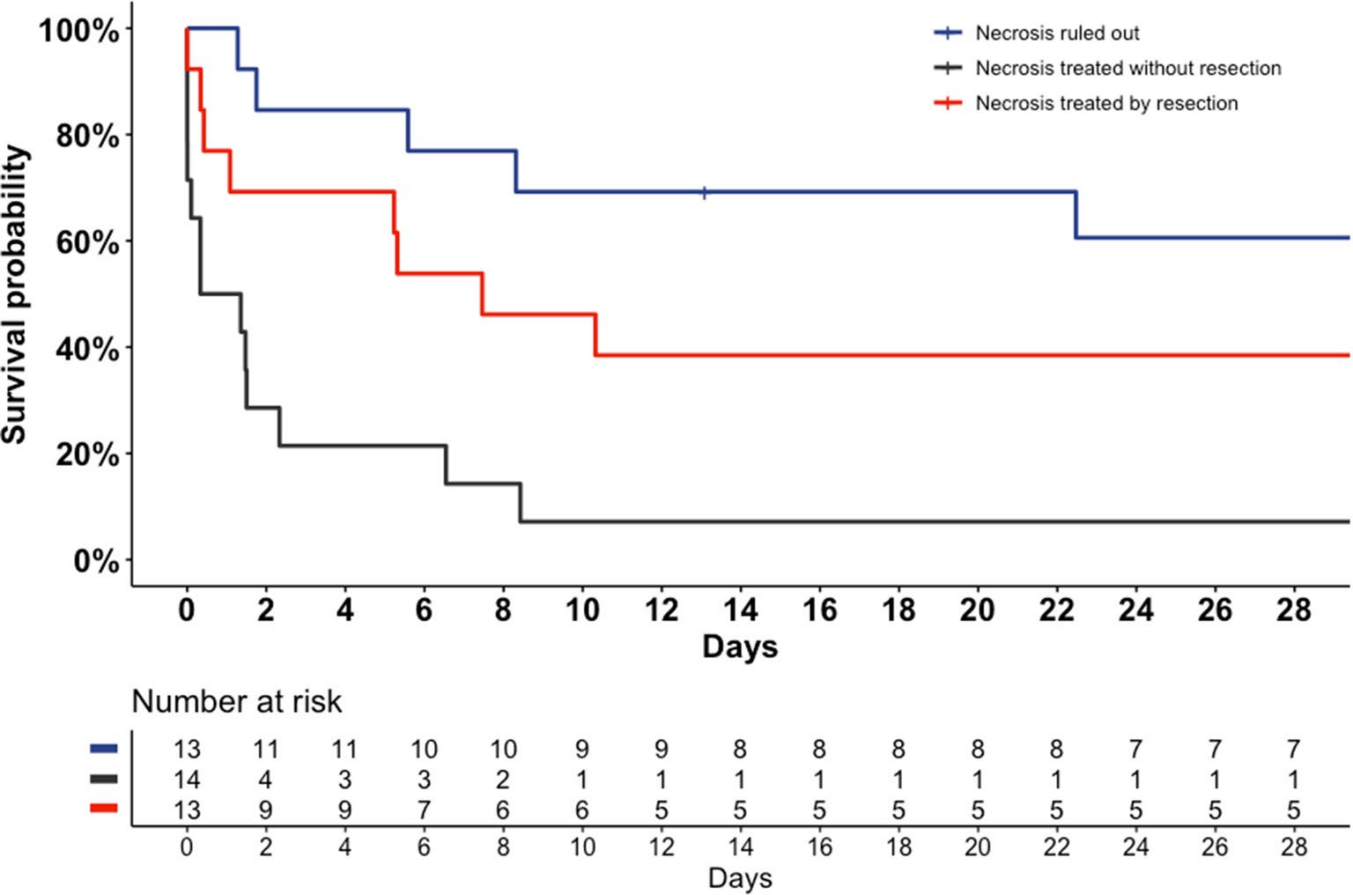
Survival plot according to intestinal necrosis presence and necrosis resection

Among 27 patients with intestinal necrosis, 13 (46.4%) underwent necrosis resection. Intestinal resection was significantly associated with ICU survival (38.5%), whereas no patient survived without necrosis resection (HR = 0.31 [0.12–0.75], *p* = 0.01) (Table 2, Fig. 3).

I-FABP was significantly higher in intestinal necrosis and NOMI non-survivors, respectively, 10384 [3120–38334] versus 2140 [2060–4092] pg/mL (HR = 1.02 [1.01–1.03], *p* = 0.02), and 10790 [3125–37266] versus 2488 [1566–4352] pg/mL (HR = 1.02 [1.01–1.03], *p* = 0.003) (Table 2, Additional file 1: Tables S4 and S5). No relationship was observed between outcome and plasma citrulline or arginine levels.

In necrosis patients, the Cox proportional hazards multivariable model retained age, lactate and abdominal distension as factors independently associated with ICU mortality.

## Discussion

In this observational prospective multicentric study, we investigated the diagnostic performance of clinical, laboratory, CT parameters and candidate biomarkers in 61 critically ill patients with NOMI suspicion. We finally diagnosed 33 intestinal ischemia including 27 intestinal necrosis, defined by stringent criteria. The results of routine work-up investigations showed poor sensitivity of clinical signs, and CT retrieved no sign of mesenteric ischemia in 30% of patients. Interestingly, plasma I-FABP at the time of NOMI suspicion presented high accuracy for necrosis prediction. Additionally, we focused on clinical, biological and management features and their association with outcomes. While no patient survived without necrosis resection, ICU survival increased up to 38.5% in patients undergoing resection of necrotic intestinal segments.

NOMI is known from long time as a challenging diagnosis and is frequently suspected in ICU in a context of a clinical worsening [1, 11]. Recently, monocentric retrospective studies highlighted several conditions affecting critically ill patients potentially promoting NOMI such as septic shock, successfully resuscitated cardiac arrest, post-cardiac surgery or cardiogenic shock [2, 4–6, 10]. These observations reinforce the hypothesis that NOMI represents the late stage of acute gastrointestinal failure process [22], preceded by still unclear pathophysiological mechanisms including impaired tissue perfusion responsible for gut barrier failure and endotoxin translocation, endothelial dysfunction and ischemia–reperfusion injury with increased local cytokine production [1, 23–25]. A working group of the ESICM recently underlined the need to validate new biomarkers and to increase the pathophysiological understanding of NOMI genesis [14]. To our knowledge, our study is the first prospective multicentric study focused on NOMI diagnosis and prognosis.

The diagnosis process should answer 2 highly important issues. First of all is to perform an early NOMI diagnosis to avoid progression to transmural necrosis [1, 16]. The second one is to dispose reliably information on the presence of intestinal necrosis to guide decision toward surgical treatment. In this study, we used stringent criteria to evaluate the diagnosis features accuracy according to the presence of necrosis or ischemia. Confirming previously published data [4], clinical digestive signs lacked sensitivity and specificity in performing NOMI diagnosis. In the same way, the increase of routine laboratory markers reflecting tissue ischemia lacked specificity, but should reinforce NOMI suspicion in patients at risk [17, 26]. In our cohort, CT signs of mesenteric ischemia could also not differentiate intestinal necrosis from ischemia without necrosis. In the literature, bowel dilation has been proposed as a marker of transmural intestinal necrosis, being more accurate when associated with multiorgan failure and increased arterial lactate [27]. However, most of mesenteric ischemia from the cohort of Nuzzo et al. has a vascular occlusion origin, a setting with increased CT diagnosis performance. On the contrary, we already reported disappointing CT performance in the specific NOMI setting and here we observed the same findings [4], CT scan concluding to no sign of intestinal ischemia in almost one-quarter of patients with intestinal necrosis.

In this cohort, we prospectively investigated plasma I-FABP and citrulline performance in NOMI, as they represent potential promising biomarkers. In particular, experimental studies demonstrated early increase of I-FABP after gut ischemia onset [28, 29]. Comparing plasma I-FABP concentration at time of suspicion in 27 patients with definite intestinal necrosis to 13 patients with intestinal necrosis ruled out, we found an AUC of 0.83 [0.70–0.96], and proposed a threshold of 3114 pg/mL with good positive predictive value (90% [67–96]) and moderate negative predictive value (58% [36–93]). Thuiyjls et al. studied I-FABP accuracy in 22 AMI patients compared to 24 other patients with initial AMI suspicion finally ruled out [30]. In their work, urinary and plasma I-FABP AUC reached 0.93 and 0.70. However, in critical illness and particularly in NOMI, we observed a high prevalence of acute renal failure, and urine might not be available. Another study of Matsumoto et al. found an AUC of 0.88 for AMI diagnosis including 15 NOMI and 9 arterial occlusions [31]. The authors highlighted I-FABP increase in various non-vascular intestinal ischemia etiologies. Although both studies of Thuiyjls and Matsumoto suffered methodological issues concerning classification of ruled out cases of mesenteric ischemia, altogether, these results suggest I-FABP could be a reliable and early biomarker of NOMI. Importantly, I-FABP threshold for mesenteric ischemia diagnosis is not consensual [25] and its accuracy may differ according to ELISA kits [32]. While promising, plasma I-FABP integration in order to monitor intestinal ischemia is probably too early at this point and should be further explored to refine plasma I-FABP accuracy in larger cohorts.

Plasma citrulline, proposed as a marker of acute intestinal failure in critically ill patients [19], had never been studied in a NOMI cohort before. We observed no relationship between plasma citrulline levels and presence of NOMI. Furthermore, plasma citrulline levels in presence of intestinal necrosis were not associated with outcome. In the literature, low plasma citrulline in critically ill patients has been reported, and was found to be associated with clinical signs of intestinal dysfunction, bacterial translocation, elevated I-FABP and worse outcomes, suggesting rational for its use as a NOMI biomarker [19, 23, 33, 34]. Our findings could be explained by a delayed time of measurement compared to previously cited studies investigating it early after admission, and a high prevalence of acute renal failure in our cohort, which may lead to high plasma citrulline concentrations despite reduction of the enterocyte mass [20]. Our data do not support an interest of citrulline in diagnosis of late stage NOMI.

NOMI therapeutic management is based on scarce evidence in the literature. Angiography, enabling the in situ administration of a continuous infusion of vasodilatory drugs, was considered an efficient treatment for NOMI [35–37]. However, the clinical benefit of this technique is unknown at the stage of intestinal necrosis [12]. The tolerance of vasodilatory drugs in hemodynamically unstable patients is unclear and treatment relies mainly on necrotic intestinal segment resections. To our knowledge, increased survival associated with resection of necrotic intestinal segments (38.5% versus no survivors without surgical resection) has never been reported in NOMI setting before. These findings highlight the importance to improve the screening of NOMI patients who may benefit of surgery given their high expected mortality in absence of necrosis resection [38]. Importantly, our results found potential interest of I-FABP in this way, allowing diagnosis of intestinal necrosis. However, the statistical association of I-FABP with ICU mortality in presence of intestinal necrosis was not confirmed in multivariate analysis. This result could be explained by the small size of the population, powered primarily to investigate diagnosis performance. Larger studies could help to clarify the interest of I-FABP in surgical treatment decision-making for NOMI patients. Lastly, bacteremia related to intestinal translocation was observed in 30% of NOMI patients, suggesting the benefit of antibiotic regimen targeting bacteria from digestive tract. Endotoxemia related to gut barrier rupture had been observed by Grimaldi and al after cardiac arrest [23], reinforcing this finding.

While common in the field of clinical research on NOMI in critical care, our study’s limitations are mainly methodological. The pathophysiology and the time-course of gastrointestinal failure are still unprecise, may vary greatly, and definitions are lacking [14]. We have chosen to focus on NOMI, thought to represent the worst stage of gastrointestinal injury, and raising unsolved diagnosis and therapeutic issues. The low incidence of NOMI requires an appropriate selection of the study population with consideration of the pre-test probability leading us to propose inclusion criteria. These criteria for NOMI’s suspicion, based on current knowledge in the field, could be interpreted as too late, resulting in the high severity of illness at the time of diagnosis. However, a lower threshold of NOMI suspicion would have led to unjustified invasive exams. We recognize that this high pre-test probability may have resulted in the high diagnosis performance observed for plasma I-FABP. Additionally, classification of patients in which NOMI can be ruled out is challenging. Consequently, NOMI was diagnosed using stringent criteria, mainly based on macroscopic findings, increasing the validity of patients’ classification. Also, whereas abdominal distension presented an interesting trend in diagnosis performance and in prognosis value in patients who had a diagnosis of intestinal necrosis, it has to be acknowledged that abdominal distension is a non-parametric parameter subject to variability assessment. Intra-abdominal pressure measurements and abdominal compartment syndrome as defined by the World Society of Abdominal Compartment Syndrome would have provide interesting information regarding NOMI diagnosis and prognosis in the study population but were not recorded by the study centers in usual care [39]. Finally, while observing increased survival of patients with intestinal necrosis resection, the observational design of the study does not allow to conclude a causal link.

## Conclusion

In this observational prospective multicentric study involving 61 critically ill patients with NOMI suspicion, intestinal necrosis was associated with extremely high mortality, and increased survival when necrosis resection was performed. Plasma I-FABP was associated with intestinal necrosis diagnosis. On the contrary, plasma citrulline was not useful to diagnosis. Further studies are needed to investigate the performance of both biomarkers in less severe forms of NOMI and set I-FABP threshold valuable in clinical decision-making.

## Supplementary Information

**Additional file 1: Table S1. **Demographic and inclusion criteria among patients with defined and ruled out NOMI. **Table S2. **Demographic and inclusion criteria among patients with gastrointestinal failure with defined and ruled out intestinal necrosis. **Table S3. **Digestive, biological parameters and CT results among patients with defined and ruled out NOMI. **Table S4. **Description of prognosis features according to ICU survival of patients with definite intestinal necrosis. **Table S5.** Description of prognosis features according to ICU survival of patients with NOMI. **Table S6.** Cox proportional hazards model univariable analysis of ICU mortality associated factors in patients with definite NOMI. **Table S7.** Description of surgical and endoscopic findings for patients with definite necrosis. **Table S8. **Amount of missing data for each biological variable.

## Data Availability

The data sets used and/or analyzed during the current study are available from the corresponding author on reasonable request.

## Abbreviations

CT: Computed tomography
ECMO: Extracorporeal membrane of oxygenation
ICU: Intensive care unit
I-FABP: Intestinal-fatty acid binding protein
IQR: Interquartile range
NOMI: Non-occlusive mesenteric ischemia
SAPS: Simplified acute physiology score
SOFA: Sequential organ-failure assessment
