# Supplementary material for: A multicentric prospective observational study of diagnosis and prognosis features in ICU mesenteric ischemia: the DIAGOMI study
Source: Ann Intensive Care. 2022 Dec 17;12:113. doi: 10.1186/s13613-022-01092-8 (PMC9759607; doi:10.1186/s13613-022-01092-8)
Supplement: Supplementary file 1 — Additional file 1: Table S1. Demographic and inclusion criteria among patients with defined and ruled out NOMI. Table S2. Demographic and inclusion criteria among patients with gastrointestinal failure with defined and ruled out intestinal necrosis. Table S3. Digestive, biological parameters and CT results among patients with defined and ruled out NOMI. Table S4. Description of prognosis features according to ICU survival of patients with definite intestinal necrosis. Table S5. Description of prognosis features according to ICU survival of patients with NOMI. Table S6. Cox proportional hazards model univariable analysis of ICU mortality associated factors in patients with definite NOMI. Table S7. Description of surgical and endoscopic findings for patients with definite necrosis. Table S8. Amount of missing data for each biological variable. [file 13613_2022_1092_MOESM1_ESM.pdf]

A multicentric prospective observational study of diagnosis and prognosis features in ICU mesenteric ischemia: the DIAGOMI study

Simon Bourcier, Guillaume Ulmann, Matthieu Jamme, Guillaume Savary, Marine Paul, Sarah Benghanem, Jean-Rémi Lavillegrand, Matthieu Schmidt, Charles-Edouard Luyt, Eric Maury, Alain Combes, Frédéric Pène, Nathalie Neveu, Alain Cariou

Additional file

Inclusion and Exclusion criteria

Criteria defining NOMI and Necrosis Diagnosis

Table S1. Demographic and inclusion criteria among patients with defined and ruled out NOMI

Table S2. Demographic and inclusion criteria among patients with gastrointestinal failure with defined and ruled out intestinal necrosis

Table S3. Digestive, biological parameters and CT results among patients with defined and ruled out NOMI

Table S4. Description of prognosis features according to ICU survival of patients with definite intestinal necrosis

Table S5. Description of prognosis features according to ICU survival of patients with NOMI

Table S6. Cox proportional hazards model univariable analysis of ICU-mortality associated factors in patients with definite NOMI

Table S7. Description of surgical and endoscopic findings for patients with definite necrosis

Table S8. Amount of missing data for each biological variable

Inclusion and Exclusion criteria

Patients were enrolled in the study if NOMI was clinically suspected by physicians in charge and based on prespecified inclusion criteria and absence of exclusion criteria. The presence of at least two **inclusion criteria** was necessary among:

- A new-onset or worsening circulatory failure requiring catecholamines
- Digestive signs of gastrointestinal dysfunction(22): abdominal pain, diarrhea, upper or lower digestive hemorrhage, vomiting or gastric residual>300ml, abdominal distension
- Biological signs evoking tissue ischemia or acute cell lysis: arterial lactate value > 2 mmol/l, increase of serum LDH, creatin kinase or aspartate transaminase.
- Contrast enhanced abdominal CT-scan signs of mesenteric ischemia: bowel-wall thickening or thinning with distension, lack of parietal enhancement, pneumatosis intestinalis and portal venous gas.

Patients were not involved in the study if they had surgical past of extensive bowel resection, an obvious differential diagnosis explaining the clinical worsening or a withholding for the diagnosis and therapeutic workup (endoscopy and laparoscopy with surgical bowel resection).

Criteria defining NOMI and Necrosis Diagnosis

<table>
<tr><th>Diagnosis of NOMI if one criteria among</th><th>Exclusion of NOMI</th></tr>
<tr><td>CT scan signs of mesenteric ischemia:
• Bowel-wall thickening / thinning with distension
• Lack of parietal enhancement
• Parietal pneumatosis
• Portal gas</td><td rowspan="3">Two negative investigations among CT scan, gastrointestinal endoscopy or surgery</td></tr>
<tr><td>Gastrointestinal endoscopy grade 1, 2 or 3
• Grade 1: mucosal oedema and erythema
• Grade 2: non-necrotic ulcerations on an oedematous mucosa
• Grade 3: necrosis with grey-black mucosal discoloration</td></tr>
<tr><td>Surgery with macroscopic findings of mesenteric ischemia</td></tr>
</table>

<table>
<tr><th>Diagnosis of Intestinal Necrosis if one criteria among</th><th>Exclusion of Intestinal Necrosis</th></tr>
<tr><td>Gastrointestinal endoscopy: necrosis with grey-black mucosal discoloration (grade 3)</td><td rowspan="2">Two negative investigations for the presence of necrosis among CT scan, gastrointestinal endoscopy or surgery</td></tr>
<tr><td>Surgery with macroscopic findings of intestinal necrosis</td></tr>
</table>

Table S1. Demographic and inclusion criteria among patients with defined and ruled out NOMI

Characteristics	Total (*n*=61)	Ruled out NOMI (*n*=8)	Definite NOMI (*n*=33)	*P* value
**At ICU admission**				
Age (years)	63.5 [56.5–68.9]	65.9 [58.8–73.0]	60.7 [56.0–67.6]	0.41
SAPS II	71 [54–77]	54 [46–76]	71 [55–85]	0.24
Males	38 (62.3)	5 (62.5)	19 (57.6)	1
Body mass index (kg/m^2)	27.2 [23.8–29.4]	26.6 [23.7–28.6]	27.2 [23.9–30.0]	0.59
SOFA	12 [9–16]	11 [5–15]	15 [11–17]	0.18
**Comorbidities**				
Diabetes	12 (19.7)	2 (25.0)	4 (12.1)	0.58
Hypertension	33 (54.1)	5 (62.5)	15 (45.5)	0.45
Smoking	29 (47.5)	4 (50.0)	15 (45.5)	1
Coronary disease	19 (31.1)	4 (50.0)	8 (24.2)	0.20
Peripheral arterial disease	10 (16.4)	4 (50.0)	3 (9.1)	0.02
End Stage Renal Disease	2 (3.3)	0 (0.0)	1 (3.0)	1
Atrial fibrillation	12 (19.7)	2 (25.0)	6 (18.2)	0.64
**Main diagnosis at ICU admission**				0.53
Cardiogenic shock	20 (32.8)	4 (50.0)	10 (30.3)	
Septic shock	15 (24.6)	1 (12.5)	8 (24.2)	
ARDS	10 (16.4)	1 (12.5)	7 (21.2)	
Cardiac arrest	5 (8.2)	0 (0.0)	3 (9.1)	
Hypovolemic shock	4 (6.6)	2 (25.0)	2 (6.1)	
Other	7 (11.5)	0 (0.0)	3 (9.1)	
**At inclusion**				
Time to inclusion (Days)	5.7 [1.6–10.6]	4.1 [2.1–6.8]	5 .9 [1.6–10.5]	0.41
SOFA at inclusion	16 [11–19]	13 [9–16]	18 [14–20]	0.06
Norepinephrin dose (μg/Kg/mn)	0.72 [0.20–1.93]	0.34 [0.18–0.51]	1.39 [0.44–1.93]	0.02

Venous arterial ECMO	21 (34.4)	2 (25.0)	15 (45.5)	0.43
Mechanical ventilation	51 (83.6)	6 (75.0)	29 (87.9)	0.58
RRT	43 (70.5)	4 (50.0)	26 (78.8)	0.18
**Nutrition route last 24 hours**				1
0	40 (65.6)	5 (62.5)	20 (60.6)	
Enteral	19 (31.1)	3 (37.5)	12 (36.4)	
Parenteral	2 (3.3)	0 (0.0)	1 (3.0)	
**Number of inclusion criteria**				0.08
2	21 (34.4)	4 (50.0)	6 (18.2)	
3	29 (47.5)	4 (50.0)	16 (48.5)	
4	11 (18.0)	0 (0.0)	11 (33.3)	
**Inclusion criteria**				
Digestive manifestations	47 (77.0)	8 (100)	28 (84.8)	0.56
Shock or worsening of shock	55 (90.2)	6 (75.0)	30 (90.9)	0.25
Biological signs	56 (91.8)	6 (75.0)	32 (97.0)	0.09
CT signs	15 (24.6)	0 (0.0)	14 (42.4)	0.04
**Time between Inclusion and Diagnosis (Hours)**	–	13.4 [4.1–22.8]	4.1 [0.0–25.4]	0.37

Results are expressed as median [interquartile range] or *n* (%). SAPS II = Simplified Acute Physiology Score II, ICU = Intensive Care Unit, SOFA = Sequential Organ-Failure Assessment, ARDS = Acute Respiratory Distress Syndrome, RRT = Renal Replacement Therapy, CT = Computed Tomography

Table S2. Demographic and inclusion criteria among patients with gastrointestinal failure with defined and ruled out intestinal necrosis

Characteristics	Gastrointestinal failure (*n*=54)	Ruled out necrosis (*n*=13)	Definite necrosis (*n*=27)	*P* value*
**At ICU admission**				
Age (years)	60.6 [56.4–68.0]	63.8 (59.2–72.2)	60.7 (55.8–67.6)	0.48
SAPS II	71 [53–81]	54 [47–77]	72 [55–85]	0.23

Males	32 (59.3)	9 (69.2)	14 (51.9)	0.33
Body mass index (kg/m^2)	27.0 [23.8–29.4]	24.5 [23.8–28.2]	27.2 [24.8–30.1]	0.09
SOFA	12 [9–16]	14 [5–16]	15 [11–18]	0.26
**Comorbidities**				
Diabetes	9 (16.7)	2 (15.4)	3 (11.1)	1
Hypertension	29 (53.7)	6 (46.2)	13 (48.1)	1
Smoking	27 (50.0)	6 (46.2)	12 (44.4)	1
Coronary disease	17 (31.5)	5 (38.5)	7 (25.9)	0.48
Peripheral arterial disease	9 (16.7)	4 (30.8)	3 (11.1)	0.19
End Stage Renal Disease	1 (1.9)	0 (0.0)	1 (3.7)	1
Atrial fibrillation	10 (18.5)	2 (15.4)	5 (18.5)	1
**Main diagnosis at ICU admission**				0.71
Cardiogenic shock	18 (33.3)	5 (38.4)	8 (29.6)	
Septic shock	11 (20.4)	1 (7.7)	6 (22.2)	
ARDS	9 (16.7)	2 (15.4)	4 (14.8)	
Cardiac arrest	5 (9.3)	2 (15.4)	1 (3.7)	
Hypovolemic shock	4 (7.4)	2 (15.4)	2 (7.4)	
Other	7 (13.0)	1 (7.7)	2 (7.4)	
**At inclusion**				
Time to inclusion (Days)	5.7 [1.6–10.3]	2.7 [0.9–6.5]	7.0 [2.7–10.7]	0.03
SOFA at inclusion	16 [10–20]	14 [9–18]	18 [16–20]	0.01
Norepinephrin (μg/Kg/mn)	0.65 [0.19–1.83]	0.46 [0.24–0.87]	1.54 [0.51–2.20]	0.02
Venous arterial ECMO	19 (35.2)	3 (23.1)	14 (51.9)	0.10
Mechanical ventilation	44 (81.5)	10 (76.9)	25 (92.6)	0.31
RRT	36 (66.7)	7 (53.8)	23 (85.2)	0.05
**Nutrition route last 24 hours**				0.44
None	34 (63.0)	8 (61.5)	16 (60.7)	
Enteral	18 (33.3)	4 (30.8)	11 (39.3)	
Parenteral	2 (3.7)	1 (7.7)	0 (0.0)	
**Number of inclusion criteria**				0.02

2	15 (27.8)	5 (38.5)	5 (18.5)	
3	28 (51.9)	8 (61.5)	11 (40.7)	
4	11 (20.4)	0 (0.0)	11 (40.7)	
**Inclusion criteria**				
Digestive manifestations	47 (87.0)	12 (92.3)	23 (85.2)	1
Shock or worsening of shock	48 (88.9)	10 (76.9)	25 (92.6)	0.31
Biological signs	49 (90.7)	11 (84.6)	26 (96.3)	0.24
CT signs	16 (29.6)	1 (7.7)	14 (51.9)	0.006
**Time between Inclusion and Certainty Diagnosis (Hours)**	–	20.6 [7.5–35]	2.8 [0.0–13.9]	0.02

Results are expressed as median [interquartile range] or *n* (%). **P*-value is presented for statistical comparison of ruled out and definite necrosis. SAPS II = Simplified Acute Physiology Score II, ICU = Intensive Care Unit, SOFA = Sequential Organ-Failure Assessment, ARDS = Acute Respiratory Distress Syndrome, RRT = Renal Replacement Therapy, CT = Computed Tomography

Table S3. Digestive, biological parameters and CT results among patients with defined and ruled out NOMI

**Characteristics**	**Total (*n*=61)**	**Ruled out NOMI (*n*=8)**	**Definite NOMI (*n*=33)**	***P* value**
**Digestive manifestation criteria**	47 (77.0)	8 (100)	28 (84.8)	0.56
Upper digestive hemorrhage	4 (6.6)	1 (12.5)	2 (6.1)	0.49
Vomiting, residual gastric volume*	24 (39.3)	3 (37.5)	15 (45.5)	1
Lower digestive hemorrhage	15 (24.6)	5 (62.5)	10 (30.3)	0.12
Diarrhea	31 (50.8)	5 (62.5)	16 (48.5)	0.69
Abdominal pain	22 (36.1)	2 (25.0)	12 (36.4)	0.69
Abdominal distension	17 (27.9)	0 (0.0)	15 (45.5)	0.02
**Worst 24 hr biological findings prior inclusion**				

Leukocyte count (G/L)	18.7 [9.9–26.2]	10.1 [9.4–15.4]	19.2 [9.5–25.0]	0.43
Platelets (G/L)	108 [60–214]	134 [104–219]	82 [44–160]	0.11
Arterial lactate, mmol/L	5.9 [3.0–10.3]	2.8 [1.0–4.2]	6.8 [4.1–12.8]	0.005
Arterial pH	7.32 [7.22–7.42]	7.44 [7.28–7.49]	7.32 [7.14–7.40]	0.02
Bicarbonates, mmol/L	19.0 [13.4–21.6]	20.9 [19.7–23.5]	15.7 [11.6–19.9]	0.006
Potassium, mmol/L	4.6 [3.9–5.3]	4.1 [3.5–4.8]	4.7 [4.4–5.4]	0.06
Aspartate transaminase (IU/L)	200 [66–836]	58 [41–102]	266 [103–1183]	0.03
Alanine transaminase (IU/L)	130 [51–492]	52 [33–97]	157 [90–768]	0.04
Bilirubine (µmol/L)	44 [14–128]	21 [16–43]	61 [14–142]	0.46
LDH (IU/L)	805 [452–2380]	402 [360–549]	1372 [601–3329]	0.006
CPK (IU/L)	676 [150–2391]	377 [74–638]	1734 [444–6044]	0.03
Procalcitonin (µg/L)	6.4 [2.0–17.2]	2.5 [0.9–6.8]	9.4 [4.6–24.9]	0.15
**Positive blood culture**	18 (29.5)	1 (12.5)	10 (30.3)	0.41
**Candidemia**	5 (8.2)	0 (0.0)	3 (9.1)	1
**Specific Biomarkers**				
Plasma I-FABP (pg/mL)	3136 [1160–13034]	1070 [748–1234]	6110 [2060–24243]	0.002
Plasma citrulline (µmol/mL)	22 [14–35]	21 [12–42]	22 [16–30]	0.70
Plasma arginine (µmol/mL)	43 [30–66]	57 [42–63]	40 [29–76]	0.33
Plasma citrulline/arginine ratio	0.47 [0.27–0.69]	0.34 [0.19–0.71]	0.49 [0.38–0.66]	0.36
**CT findings**				
Abnormal wall enhancement	15 (30.0)	0 (0.0)	13 (50)	0.01
Pneumatosis intestinalis	5 (10.0)	0 (0.0)	4 (15.4)	0.55
Bowel dilation	17 (34.0)	0 (0.0)	14 (53.8)	0.01
Portal venous gas	2 (4.0)	0 (0.0)	2 (7.7)	1
Atherosclerosis of mesenteric arteries	16 (32.0)	3 (37.5)	7 (26.9)	0.66

Results are expressed as median [interquartile range] or *n* (%). *Residual gastric volume was considered if ≥300mL. CT = Computed Tomography

Table S4. Description of prognosis features according to ICU survival of patients with definite intestinal necrosis

Characteristics of Necrosis Population	Non-survivors (n=22)	Survivors (n=5)	*P* value
Age (years)	64 [57–68]	52 [46–56]	0.11
SOFA at day of suspicion	19 [16–20]	17 [17–20]	0.94
Time between ICU admission and inclusion (Days)	6.3 [2.7–10.3]	17.5 [9.1–17.9]	0.15
Time between Inclusion and Diagnosis (Hours)	1.3 [0.0–15.8]	4.1 [2.8–9.5]	0.40
**Biological parameters**			
Lactate (mmol/L)	6.8 [4.2–14.1]	6.3 [3.5–7.3]	0.18
LDH (IU/L)	2289 [877–5716]	1053 [601–1307]	0.17
CPK (IU/L)	1881 [1041–9354]	864 [493–1538]	0.31
Aspartate transaminase (IU/L)	389 [104–1799]	220 [159–767]	0.69
Procalcitonin (µg/L)	11.1 [5.2–24.4]	11.9 [8.6–16.3]	0.86
**Positive blood cultures**	6 (27.3)	2 (40)	0.62
**Specific biomarkers**			
Plasma I-FABP (pg/mL)	10384 [3120–38334]	2140 [2060–4092]	0.01
Plasma I-FABP in patients with laparotomy	6022 [2073–35613]	2140 [2060–4092]	0.07
Plasma I-FABP in patients with resection	14201 [2767–40820]	2140 [2060–4092]	0.07
Citrulline (µmol/mL)	19 [15–33]	18 [18–24]	0.76
Arginine (µmol/mL)	47 [29–77]	36 [31–43]	0.34
Plasma citrulline/arginine ratio	0.45 [0.28–0.65]	0.56 [0.50–0.57]	0.54
**Organ supports at inclusion**			
Norepinephrin (µg/Kg/mn)	1.7 [0.7–2.5]	0.1 [0.0–1.7]	0.05
Venous arterial ECMO	11 (50)	3 (60.0)	0.68
Mechanical ventilation	18 (94.7)	4 (80.0)	1
RRT	18 (94.7)	4 (80.0)	1
**Surgical treatment**			
Resection	8/22 (36.4)	5/5 (100)	0.02
Resection delay from suspicion (h)	11.7 [4.8–82.9]	8.2 [2.8–9.5]	0.1

Results are expressed as median [interquartile range] or *n* (%). SOFA = Sequential Organ-Failure Assessment, RRT = Renal Replacement Therapy

Table S5. Description of prognosis features according to ICU survival of patients with NOMI

Characteristics of Ischemia Population	Non-survivors (n=23)	Survivors (n=10)	*P* value
Age (years)	63 [56–68]	59 [53–71]	0.80
SAPS 2	74 [54–88]	62 [55–79]	0.43
Time between ICU admission and inclusion (Days)	5.9 [2.2–10.0]	5.9 [0.7–15.5]	0.93
SOFA at inclusion	19 [16–20]	16 [11–19]	0.13
**Time between Inclusion and Diagnosis (Hours)**	2.1 [0.0–21.9]	10.3 [3.2–24.8]	0.25
**Biomarkers**			
Plasma I-FABP (pg/mL)	10790 [3125–37266]	2488 [1566–4352]	0.04
Plasma citrulline (µmol/L)	20 [15–32]	24 [18–29]	0.91
Plasma arginine (µmol/L)	43 [30–76]	38 [30–49]	0.34
Plasma citrulline/arginine ratio	0.45 [0.30–0.62]	0.56 [0.43–0.70]	0.65
**Necrosis**			
None	1 (4.3)	4 (40.0)	0.02
Yes	22 (95.7)	6 (60.0)	
**Organ supports**			
Norepinephrin dose (µg/Kg/mn)	1.54 [0.75–2.46]	0.53 [0.03–1.59]	0.03
Venous arterial ECMO	11 (47.8)	4 (40.0)	0.72
Mechanical ventilation	22 (95.7)	7 (70.0)	0.07
RRT	20 (87.0)	6 (60.0)	0.16

Results are expressed as median [interquartile range] or *n* (%). SAPS II = Simplified Acute Physiology Score II, SOFA = Sequential Organ-Failure Assessment, RRT = Renal Replacement Therapy

Table S6. Cox proportional hazards model univariable analysis of ICU-mortality associated factors in patients with definite NOMI

Characteristics of NOMI Population	CSH	95% CI	*P* value
Age (+10 years)	1.10	0.81–1.51	0.50
SAPS 2	1.01	0.99–1.03	0.50
SOFA at inclusion	1.09	0.98–1.21	0.11
**Arterial Lactate (mmol/L) at inclusion**	1.13	1.03–1.23	0.007
**Biomarkers**			
Plasma I-FABP (pg/mL, +1000 units)	1.02	1.01–1.03	0.003
Plasma citrulline (μmol/L)	1.0	0.97–1.03	0.91
Plasma arginine (μmol/L)	1.01	1.0–1.02	0.11
Plasma citrulline/arginine ratio	1.06	0.51–2.22	0.90
**Necrosis**	6.45	0.87–48.0	0.07
**Organ supports**			
Norepinephrin dose (μg/Kg/mn)	1.31	1.11–1.56	0.002
Mechanical ventilation	5.14	0.69–38.3	0.11
RRT	2.36	0.70–7.97	0.20

CSH = Cause-Specific Hazard ratio, SAPS II = Simplified Acute Physiology Score II, SOFA = Sequential Organ-Failure Assessment, RRT = Renal Replacement Therapy

Table S7. Description of surgical and endoscopic findings for patients with gastrointestinal necrosis

Findings	N (%)
**Necrosis location***	
Stomach and/or duodenum	6 (25)
Jejunum and/or ileum	10 (42)
Right colon	11 (46)
Left colon	16 (67)
Rectum	12 (50)
**Extensive (≥2 intestinal segments)**	17 (71)

**Peritonitis**	8 (30)

*Necrosis location was missing for 3 patients leading to present frequencies of necrosis location for 24 patients

Table S8. Missing values

**Characteristics**	**Total (*n*=61)**	**Ruled out necrosis**	**Definite necrosis**	**Ruled out Ischemia**	**Definite Ischemia**
**Worst 24 hr prior inclusion biological findings**					
Leukocyte count (G/L)	0	0	0	0	0
Platelets (G/L)	0	0	0	0	0
Arterial lactate, mmol/L	0	0	0	0	0
Arterial pH	0	0	0	0	0
Bicarbonates, mmol/L	0	0	0	0	0
Potassium, mmol/L	0	0	0	0	0
Aspartate transaminase (IU/L)	0	0	0	0	0
Alanine transaminase (IU/L)	0	0	0	0	0
Bilirubine (µmol/L)	0	0	0	0	0
LDH (IU/L)	5	0	4	0	4
CPK (IU/L)	8	0	6	0	6
Procalcitonin (µg/L)	13	4	4	4	4
**Positive blood culture**	0	0	0	0	0
**Candidemia**	0	0	0	0	0
**Specific Biomarkers**					
Plasma I-FABP (pg/mL)	0	0	0	0	0
Plasma citrulline (µmol/mL)	0	0	0	0	0
Plasma arginine (µmol/mL)	0	0	0	0	0
